# Investigation of the Mechanical and Optical Properties of ABS Plus Materials in Different Colors After Aging

**DOI:** 10.3390/polym17212940

**Published:** 2025-11-03

**Authors:** Muhammet Akyol, Nergizhan Anaç, Oğuz Koçar, Erhan Baysal, İrfan Akgül

**Affiliations:** 1 Department of Mechanical Engineering, Faculty of Engineering, Zonguldak Bülent Ecevit University, Zonguldak 67100, Türkiyeoguz.kocar@yahoo.com.tr (O.K.); 2 Alaplı Vocational School, Zonguldak Bülent Ecevit University, Zonguldak 67850, Türkiye; erhanbaysal@beun.edu.tr; 3 Scientific and Technological Research Center, Duzce University, Duzce 81620, Türkiye; irfanakgul@duzce.edu.tr

**Keywords:** 3D printing, ABS plus, aging, mechanical properties, optical properties

## Abstract

As the global 3D printing market continues to grow, the consumption of plastic products produced by 3D printers is also increasing. The role of 3D-printed products in both daily use and industrial applications has been progressively reinforced. Plastic materials undergo physical and chemical changes when exposed to environmental conditions such as temperature, light, and humidity. Consequently, they are subjected to aging during use, which shortens their service life. With the expanding use of 3D printing technology in various sectors such as healthcare, automotive, aerospace, and defense, it has become increasingly important to understand the changes (potential decreases or losses) in the performance of these materials after long-term exposure to environmental conditions. This study aims to contribute to the understanding of potential changes in 3D-printed ABS Plus material by examining the phenomenon of aging induced by exposure to radiation from a xenon arc lamp. ABS Plus samples of different colors (yellow, purple, red, green, and blue) were subjected to aging for 0, 112, 225, 337, and 450 h using a xenon arc lamp. To investigate the effects of aging, the mechanical (tensile, flexural, and hardness) and optical (color and gloss variations) properties of the samples were compared before and after aging. Following the mechanical tests, the fracture modes of the specimens were also examined. In addition, Scanning Electron Microscope (SEM) images were obtained to further discuss the effects of aging. The results revealed that the mechanical properties of the reference samples varied depending on color. The highest tensile strength was observed in the yellow samples (33.46 MPa), while the highest flexural strength was recorded in the green samples (58.46 MPa). After aging, the lowest tensile strength was found in the purple samples aged for 337 h (24.63 MPa), whereas the lowest bending force was measured in the red samples aged for 450 h (45.27 N). Overall, the mechanical properties of the samples varied with aging duration, with the blue and green specimens being the least affected. For the blue specimens, after 112, 225, and 337 h of aging, an increase in tensile strength was observed (2.77%, 10.54%, and 9.58%, respectively), while a decrease occurred after 450 h of aging (−6.22%). For the green specimens, after 112, 225, and 337 h of aging, the tensile strength remained similar to that of the reference sample (−2.97%, 0.23%, and 0.05%, respectively) but decreased after 445 h of aging (−8.09%). In terms of optical properties, the most significant color change (−23.51) was observed in the purple samples. Gloss measurements indicated that the impact of aging increased with exposure time.

## 1. Introduction

Since their discovery, plastic materials have been considered indispensable in both daily life and industrial applications due to their cost-effectiveness and lightweight advantages. In the late 1980s, various additive manufacturing methods (3D printing techniques), in which materials are gradually deposited layer by layer to form three-dimensional objects, emerged. Among these, Fused Filament Fabrication (FFF) has become one of the most widely used techniques because of its ease of use and versatility. With the advancement of 3D printing technology, the production and consumption of plastic feedstock materials for 3D printers have increased rapidly. Over time, the operation of 3D printers has evolved from desktop devices to professional and large-scale industrial machines, enabling the fabrication of complex and large-sized designs that are difficult or even impossible to produce using traditional methods. Nevertheless, regardless of the manufacturing technique, the degradation or aging of plastic materials occurs through the scission of molecular chains [1].

The aging of plastic materials causes changes over time in properties such as appearance, color, durability, and strength. [2]. Aging is a complex process involving multiple variables. The aging behavior of polymers under different environmental conditions has been extensively investigated to date [3]. Although numerous studies on the aging of plastics are available in the literature, research focusing on the aging of 3D-printed parts remains limited. It is well known that the mechanical properties of 3D-printed components vary depending on printing parameters such as infill density, layer thickness, build orientation, raster angle, and infill pattern. This flexible aspect of 3D printing diversifies the properties of the products and expands the range of possible material combinations. From this perspective, the effects of different extreme environmental conditions on the material properties (e.g., mechanical and optical) of 3D-printed parts constitute a relatively underexplored area [4]. Among the most widely used plastic materials in 3D printing is acrylonitrile butadiene styrene (ABS) [5,6]. Due to its properties such as high temperature resistance, impact resistance, hardness, and strength, ABS is preferred in applications requiring durable and flexible parts [7]. ABS has served as a ‘bridge’ between commodity plastics and higher-performance engineering thermoplastics and has become the best-selling engineering thermoplastic [8]. For this reason, the aging behavior of 3D-printed ABS plastics is of particular interest.

In a study investigating the photo-oxidative degradation of ABS polymer under outdoor and accelerated weathering conditions [9], the correlation between the two exposure environments was examined. Fourier Transform Infrared (FTIR) spectroscopy and mechanical property measurements were conducted to assess the effects of photo-oxidation. The results, which showed good agreement, indicated that exposure to a filtered xenon lamp (wavelength range 300–400 nm) for 1260 h at 48 °C and 50% relative humidity produced effects comparable to those of one year of outdoor exposure in Lisbon. In almost all accelerated polymer aging processes, radiation generated by different lamps—such as xenon, metal halide, fluorescent tubes, and mercury arc—is used to help catalyze the degradation process [10]. Among these, xenon arc technology is considered the most widely used method for testing the weathering resistance of polymers [11]. Danilov et al. [12] investigated the effects of environmental aging on 3D-printed ABS produced using Fused Filament Fabrication (FFF) and Digital Light Processing (DLP) techniques. The materials, in filament form (for FFF) and resin form (for DLP), were exposed to UV light, humidity, and temperature fluctuations for two months. The results revealed notable differences in mechanical strength and structural stability between ABS filament printed via FFF and ABS resin printed via DLP under aging conditions. While the ABS filament exhibited superior mechanical properties by maintaining its strength over time, the ABS resin showed significant degradation shortly after printing. M. Reza Khosravani et al. [13] analyzed the effects of accelerated aging (240 h) on 3D-printed parts produced from ABS and Acrylonitrile Styrene Acrylate (ASA) with 30% infill density, using tensile, bending, and compression tests. The test specimens exhibited improved hardness and durability under thermal aging conditions.

Amza et al. [14] analyzed the effects of accelerated aging induced by 24 h UV-C exposure on the mechanical properties of 3D-printed parts made of polycarbonate and ABS polymer (ABS-PC). The specimens were compared with a control group in terms of changes in tensile strength and compressive strength. The results showed that mechanical property degradation after irradiation was minimal, and the tensile strength of the irradiated parts was statistically equivalent to that of the control group. The irradiated specimens exhibited small reductions in hardness (5.2%) and compressive strength (6.5%). Dimensional inspections were also performed on the parts before and after accelerated aging under UV-C exposure, and no statistically significant dimensional changes were reported after irradiation.

The literature review has shown that there is limited information explaining the influence of environmental factors on the property changes of 3D-printed materials. 3D printing is becoming increasingly important. Understanding the materials used in 3D printing and the effects of working conditions on these materials can help users make better material selections. This, in turn, can enhance the performance of 3D-printed products for outdoor applications. The objective of this study is to evaluate the post-aging performance of 3D-printed ABS-based materials for specific applications, particularly outdoor use.

In this study, ABS Plus specimens of different colors (yellow, blue, purple, red, and green), produced by FFF, were subjected to accelerated aging under a xenon arc lamp for different durations (112, 225, 337, and 450 h). Mechanical tests as well as color and gloss measurements were conducted on the samples before and after aging. The fracture surfaces and test results of the parts were analyzed to evaluate the material properties. The results of this research will provide insights into both the mechanical and optical changes that occur in 3D-printed ABS-based materials and products when exposed to outdoor environmental conditions.

## 2. Material and Method

### 2.1. Aim and Concept of the Research

**Figure 1:**
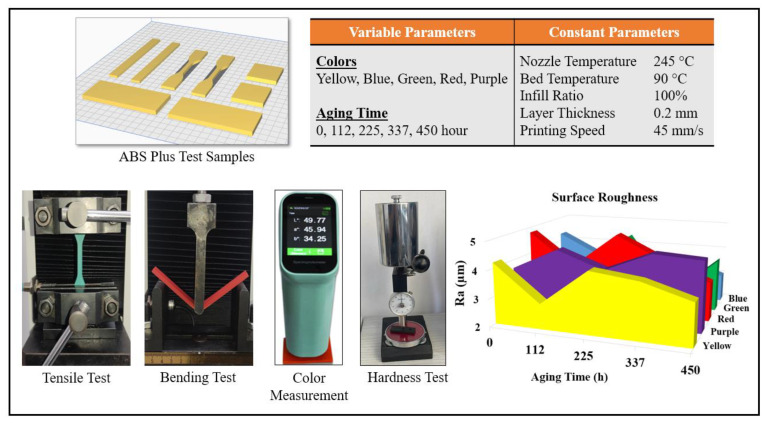
3D printing and testing.

In this study, it was aimed to investigate the effects of accelerated aging on the mechanical and optical properties of ABS Plus specimens produced using the FFF method. ABS Plus filaments in five different colors (yellow, purple, red, green, and blue) were subjected to aging for different durations (112, 225, 337, and 450 h). Before and after the aging process, tensile, bending, and hardness tests were applied to the specimens, and changes in color and gloss were examined. The results obtained were evaluated to interpret the relationship between filament color and mechanical and optical properties. While the amount of material used in the FFF method has increased day by day, users show interest in different colors and new materials. In line with this demand, filament manufacturers have been continuously conducting material development studies. With this study, it was also emphasized to users that the choice of color is important depending on the intended use of the final product. Figure 1 presents the experimental design, 3D printing parameters, and images from the applied tests.

### 2.2. Properties of Filament

ABS filament is the second most widely used raw material in additive manufacturing with 3D printers followed by PLA [15]. ABS is made from acrylonitrile, 1,3-butadiene, and styrene monomers and is petroleum-based. It is a copolymer obtained by polymerizing styrene and acrylonitrile in polybutadiene. Styrene provides gloss and a smooth surface to the plastic, while butadiene contributes flexibility at low temperatures [16]. Therefore, the most suitable operating temperature range for ABS is between −25 °C and 60 °C. Unlike PLA, ABS is not biodegradable, but it is biocompatible [17]. Being thermoplastic, it can be melted and reshaped. However, when brought to its melting point, it may release toxic gases during printing. For this reason, printing should be carried out in a well-ventilated environment or with a closed 3D printer. Moreover, during printing, ABS is sensitive to ambient conditions, which may cause warping or detachment from the build plate. Even when using an enclosed printer, printing experience is required, or adhesives should be applied to the build plate before printing. This situation may also result in additional costs.

In this study, eSUN brand ABS Plus filament in yellow, purple, red, green, and blue colors was used. Compared to conventional ABS, ABS Plus has higher mechanical properties, lower odor, and a lower shrinkage rate. The mechanical properties of ABS Plus are presented in Table 1. While variations in mechanical properties according to printing orientation are provided by filament manufacturers in technical data sheets (TDS), no information is given regarding differences based on filament color.

It should be noted that this study was limited to a single filament brand and was conducted under accelerated laboratory aging conditions, which may not fully represent performance under real-world conditions. The filament colors used in the study were selected as primary colors and the intermediate colors obtained by combining them. Yellow, blue, and red were used as the primary colors. Secondary colors were formed by mixing two primary colors: blue and yellow were combined to produce green, while red and blue were combined to produce purple. Therefore, these two secondary colors were also included in the study.

### 2.3. 3D Printing Process

ABS Plus specimens were produced using an Ultimaker S5 model FFF-type 3D printer. The Ultimaker S5 has a build volume of 230 × 190 × 200 mm, a closed printing chamber, and an air filtration system. With its integrated air manager, the printer is able to provide suitable printing conditions for ABS Plus filament material. All specimen models were sliced using Ultimaker Cura 5.7.0 software. The specimens were printed horizontally on the build plate without support structures. During the printing process, a nozzle temperature of 245 °C, a build plate temperature of 90 °C, and a print speed of 45 mm/s were employed. In addition, all specimens were produced with 100% infill ratio and a layer thickness of 0.2 mm. The printing parameters remained constant for all specimens throughout the 3D printing process. Prior to printing, a thin layer of adhesive (Filasophia brand [19]) was applied to the glass build plate to ensure perfect adhesion between the build plate and the printed parts.

### 2.4. Aging Plan

The process of accelerated weathering has been induced by the application of a Xenon-arc lamp. The initial accelerated weathering method was outlined in EN ISO 4892-2: 2013 [20], which is a standard that details the methods of exposure to laboratory light sources. This particular method is categorized under the subheading ‘Xenon-arc lamps’ (Method A, cycle no: 1).

The test conditions for this method are as follows: The test specimens were exposed to light continuously for a period of 102 min during the dry phase and 18 min during the wet phase, with the irradiance set at 0.51 W/(m^2^ nm) at 340 nm. The temperature of the black-standard thermometer (BST) was measured to be 65 ± 3 °C, the chamber temperature was recorded as 38 ± 3 °C, and the relative humidity was determined to be 50 ± 10% in the dry phase. The device utilized for the purposes of this study was the Atlas Ci4000 Weather-o-Meter. The filter combination used for the inner and outer filters was Type S boro/Type S boro. The test specimens were coded Reference, A, B, C and D. These codes corresponded to the following exposure durations by hours, respectively: 0, 112, 225, 337 and 450. The total test duration was 450 h.

To minimize out-of-chamber inhomogeneities in light, temperature, and humidity, uniform exposure across all test specimens must be ensured. Furthermore, to guarantee high reproducibility and reliability of the test results, the shelf holding the samples in the Atlas Ci4000 Weather-Ometer weathering device was rotated at a speed of 1 rpm.

### 2.5. Determination of the Color and Gloss Scale of the Specimens

Color measurements of the samples printed on a 3D printer were made using the CIE L*a*b* color system on the CHN-SPEC CS-410 portable spectrometer device (Chnspec Technology Co., Ltd., Hangzhou, China). The measurements were conducted in accordance with ASTM D2244 [21], with four measurements taken per sample. In the CIE color system, the values in which color is expressed are determined according to three color coordinates: “L*, a*, b*”. L* (darkness-lightness), a* (greenness-redness), b* (blueness-yellowness) parameters of the test parts were measured from samples before aging, and after aging. Each measurement was taken in three repetitions, and the colors of the samples were determined based on the average of the three measurements. The color measurements were performed at room temperature. Reference color measurement values were taken from 3D printed parts that were not aged. To express the color difference of parts with a single value, ${\Delta a}^{*}$, ${\Delta b}^{*}$, ${\Delta L}^{*}$ and ${\Delta E}^{*}$ (Total color change) values were calculated using the relevant formula (1).

(1) $${\Delta E}^{*}=\sqrt{{\left({\Delta L}^{*}\right)}^{2}+{\left({\Delta a}^{*}\right)}^{2}+{\left({\Delta b}^{*}\right)}^{2}}$$

Gloss is an optical property expressed as the ratio of the intensity of light reflected from a surface to the intensity of light incident upon it. Its unit is Gloss Unit (GU). When the obtained values are less than 10 GU or greater than 70 GU, the measurement angle should be adjusted to 20° (for gloss levels above 70 GU) or 85° (for gloss levels below 10 GU) [22,23]. The gloss of the specimen surfaces was measured in triplicate at room temperature using a PCE-PGM 100 gloss meter (PCE Deutschland GmbH, Meschede, Germany) in accordance with ISO 2813:2014 [24]. Gloss tests of the specimens were performed at 20°, 60°, and 85° geometries.

### 2.6. Tests Used to Determine Mechanical Properties

**Figure 2:**
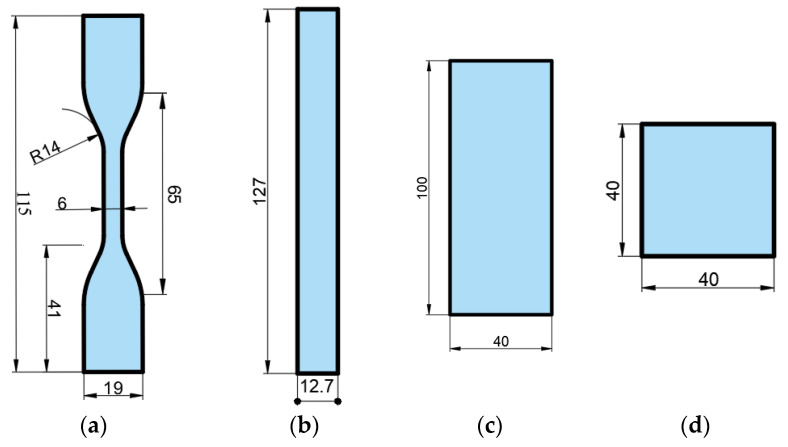
Dimensions of the specimens used for mechanical and optical tests (**a**) Tensile test (**b**) Bending test (**c**) Color, gloss, and surface roughness measurements (**d**) Specimens prepared for hardness measurement (dimensions are given in millimeters).

In the study, the dimensions of the specimens used for mechanical and optical tests are shown in Figure 2. Tensile testing (Figure 2a) was conducted in accordance with ASTM D638 [25], and three-point bending testing (Figure 2b) was performed in accordance with ASTM D790 [26]. For both tensile and bending tests, a WDW-5 universal testing machine with a 5 kN capacity was used. The tensile test was carried out at a crosshead speed of 1 mm/min, while the bending test was conducted at 5 mm/min, both at room temperature, with four repetitions. Hardness tests were performed using a Shore-D durometer (Loyka, Shenzhen Yibai Network Technology Co., Ltd., Shenzhen, China) in accordance with ASTM D2240-15 [27]. Hardness measurements were taken from ten different points on each specimen, and the average values were calculated. The specimen dimensions used for hardness testing are given in Figure 2d. The arithmetic mean surface roughness (Ra) values (µm) of the specimens were measured using a portable surface roughness tester, model SJ301 (Mitutoyo, Kawasaki, Japan) (Figure 2c). Measurements were taken five times for each specimen, and the average values were calculated. The temperature of the room where the experiments were conducted was 22 °C, and the humidity level was 59%.

## 3. Results and Discussion

### 3.1. Properties of the Base (Reference) Materials

**Figure 3:**
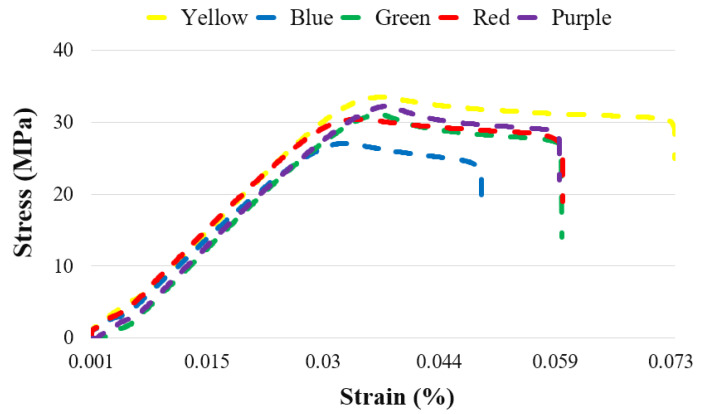
Stress–strain curves of unaged specimens in five different colors.

In Figure 3, the stress–strain curves of the unaged specimens for five different colors are presented, while Table 2 shows their ultimate tensile strength (UTS) and percentage elongation values. Among the five colors tested, the highest tensile strength was observed in the yellow specimens at 33.46 MPa. This was followed by the purple (32.20 MPa), green (31.02 MPa), red (30.39 MPa), and blue (27.03 MPa) specimens, respectively. Examination of the elongation values revealed that the highest elongation was obtained in the yellow specimens, with 7.27%.

Gao et al. investigated the effect of color on the mechanical properties of ABS and PLA materials (eSUN, Beijing, China) by using seven different colors (red, yellow, blue, green, black, orange, purple, and natural). For all specimens, a layer thickness of 0.15 mm and an infill ratio of 100% were applied as process parameters. In ABS, the highest UTS value was recorded for the red specimens at 24.9 MPa, followed by the green and yellow specimens at 24 MPa and 22 MPa, respectively. In PLA specimens, the highest UTS was observed in the purple specimens at 52.5 MPa, while the yellow and orange specimens showed values of 51.7 MPa and 50.6 MPa, respectively [28]. Frunzaverde et al. examined the effect of color (gray, red, natural, and black) and layer thickness (0.05, 0.10, 0.15, and 0.20 mm) on the mechanical properties of PLA. At all layer thicknesses, the highest tensile strength (UTS) was obtained in the gray specimens [29]. Wittbrodt et al. investigated the effects of color by using natural, black, gray, blue, and white PLA. As a result, they reported that the natural color material exhibited the highest UTS value [30].

In the literature, studies examining the relationship between color and mechanical properties have rarely focused on ABS materials, while more research has been conducted on PLA. A common finding across these studies is that the mechanical properties vary depending on the filament color. However, the color reported to have the best mechanical properties differs across studies. This variation can be attributed to the use of different filament brands in these studies. Another reason is that filament manufacturers employ different colorants to produce the desired filament colors.

**Figure 4:**
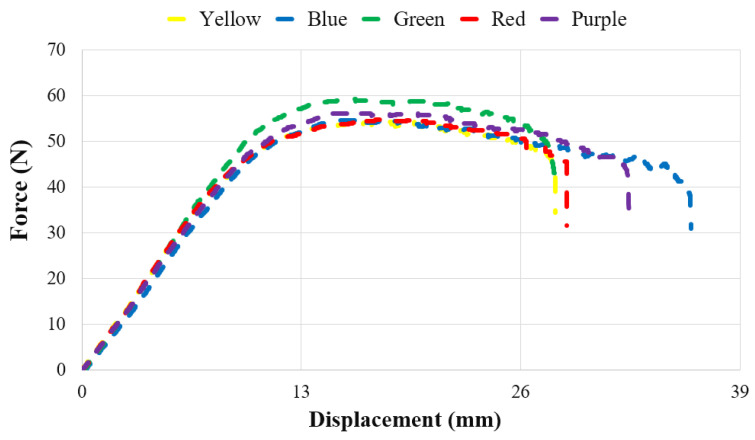
Bending test graph of the base specimens in different colors.

In Figure 4, the bending test graphs of the unaged specimens are presented, and Table 3 shows the maximum bending force and displacements recorded during the bending tests. Among the five different colors tested, the highest bending force was observed in the green specimens at 58.46 N. This was followed by the purple (56.18 N), yellow (54.88 N), blue (54.59 N), and red (54.40 N) specimens, respectively. In their study, Gao et al. reported the highest flexural strength in the green specimens at 35.6 MPa, followed by the purple specimens at 35.1 MPa [28]. The findings of Gao et al. are consistent with the results of the present study.

**Figure 5:**
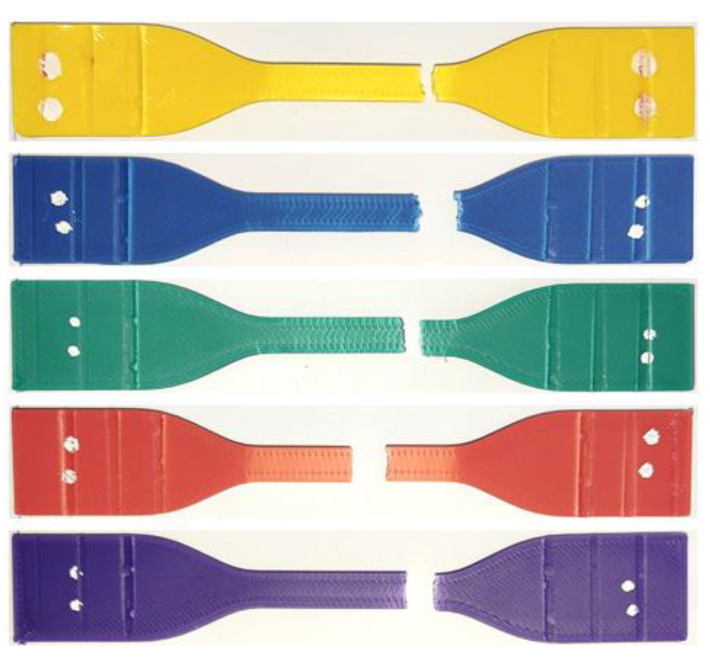
Fracture surfaces of the reference specimens after tensile testing.

Figure 5 shows the fracture surfaces of the reference specimens after tensile test. When the reference specimens are compared with one another, it can be observed that although their colors differ, the fracture shapes are similar. In the fracture region, dense lines perpendicular to the applied tensile stress are visible. In bending test sample, it is clearly seen that in the yellow and red bending specimens, the lines caused by the applied load appear as cracks in the fracture region. In contrast, in the green, purple, and blue bending specimens, although the bending occurred in the same measurement region as the other specimens, bending deformation due to stress was observed on the fracture surfaces.

### 3.2. Tensile Strength After Aging

**Figure 6:**
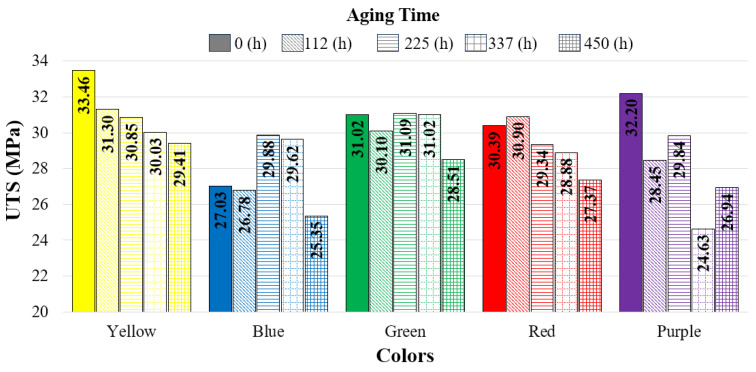
Comparison of UTS values with respect to aging duration.

Figure 6 presents the variation in UTS with aging durations, while Table 4 shows the stress–strain values corresponding to different aging durations. In the yellow specimens, UTS decreased as the aging duration increased. The lowest UTS for the yellow specimens (29.41 MPa) was recorded after 450 h of aging.

In the blue specimens, the lowest UTS was recorded at 25.35 MPa after 450 h of aging. Compared to the reference specimen (27.03 MPa), the UTS of the blue specimens increased at 225 h (29.88 MPa) and 337 h (29.62 MPa). For the green specimens, the UTS values of the reference, 112, 225, and 337 h aged specimens were found to be similar, while an 8% decrease was observed after 450 h of aging compared to the reference. In the red specimens, a 1.6% increase in UTS was observed at 112 h compared to the reference, whereas decreases occurred at the other aging durations. The greatest reduction for the red specimens was recorded after 450 h of aging (27.37 MPa), corresponding to a 9.9% decrease.

In the purple specimens, the greatest decrease in UTS was observed after 337 h of aging, with a reduction of 23.5%. Among the five colors tested, the purple specimens exhibited the most inconsistent behavior with respect to aging duration. Amza et al. conducted aging tests on ABS/PC specimens produced by 3D printing using UV-B (λ = 315 nm) and UV-C (λ = 254 nm) irradiation for 24 h. After aging, no significant change in tensile strength was observed (reference: 39.26 MPa, UV-B: 39.51 MPa, UV-C: 38.53 MPa), while compressive strength decreased by 5.2% under UV-B and 6.5% under UV-C exposure [14]. Ünal et al. subjected ABS Plus specimens in three different colors (yellow, green, and blue) and with three different infill ratios (50%, 75%, and 100%) to a 96 h salt spray test at 35 °C. As a result, they reported a slight increase in the yellow and green specimens at 100% infill ratio, while a slight decrease was observed in the blue specimens [31]. These two studies demonstrate that after 24 h (UV-B and UV-C) and 96 h (salt spray) of aging, the mechanical properties were only minimally affected. In the present study, however, it was determined that both color and aging duration had a significant effect on the variation in tensile strength.

Popescua et al. examined the aging behavior of ABS materials used in the healthcare sector under natural aging (storage environment) and repeated sterilization conditions. In their study, the samples were divided into two groups: Group 1 (control group) was subjected only to natural aging, while the samples in Group 2 were exposed to both aging and multiple sterilization processes. As a result, they reported that aging caused by natural storage and sterilization did not lead to a significant change in the mechanical properties of the materials [32].

Boldizara and Möller investigated the changes in the mechanical properties of ABS material subjected to repeated extrusion, aging, and combined extrusion/aging cycles. The aging process was carried out by keeping the samples at 90 °C for 72 h after extrusion. As a result, it was observed that with an increasing number of cycles, the elongation at break significantly decreased in the aged samples, while it increased in the extruded samples. In the samples subjected to both extrusion and aging, the elongation at break exhibited a fluctuating behavior [33].

Bergaliyeva et al. investigated the effects of thermal and hydrothermal aging on PLA materials. For thermal aging, the samples were kept at 50 °C, while for hydrothermal aging, they were exposed to 50 ± 2 °C and 70 ± 5% humidity for 8, 16, 24, 48, 72, 168, 672, and 1344 h. As a result, thermal aging led to an increase in mechanical properties in all samples compared to the reference, whereas hydrothermal aging caused a decrease in mechanical properties after 1344 h of exposure [34].

Amza et al. evaluated the effects of UVC aging on PLA and PETG. For this purpose, the printed samples were exposed to UVC for 24 h and then allowed to cool at room temperature for 4 h. By comparing the control group with the aged samples, they examined the tensile strength, compressive strength, and creep behavior. As a result, it was reported that PLA parts showed no visible changes in appearance after aging, whereas PETG samples exhibited darkening and yellowing. In terms of mechanical properties, tensile strength decreased by 9.1% in PLA and 38.1% in PETG, while compressive strength decreased by 13.1% in PLA and 33.9% in PETG [35].

A review of the literature shows that various studies have been conducted using different aging techniques and materials. These studies have investigated the effects of natural, thermal, UV, and hydrothermal aging methods on the most commonly used 3D printing materials. A common point among previous studies is that the aging duration and aging temperature [36] significantly affect material degradation, and it is necessary to evaluate the behavior of parts under outdoor environmental conditions.

**Figure 7:**
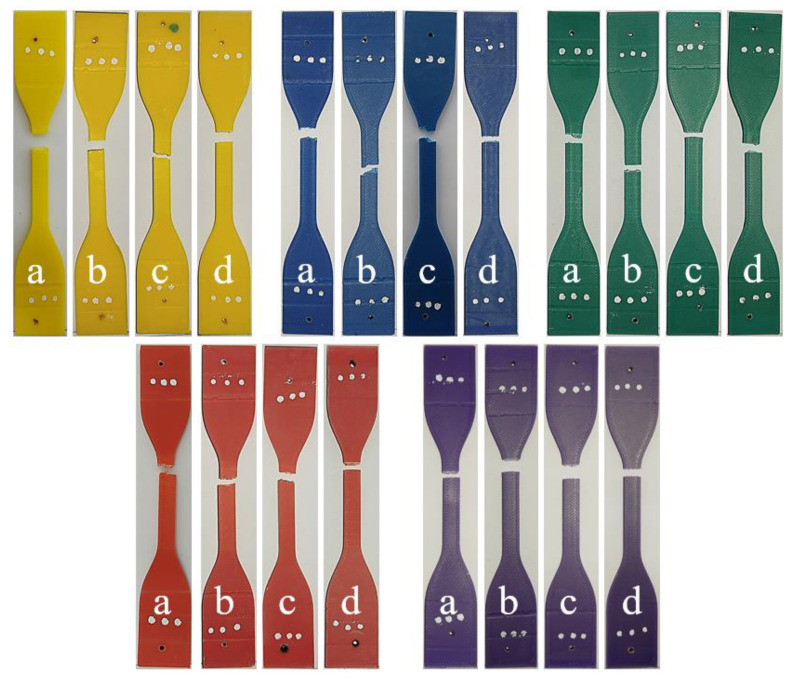
Fracture images of tensile specimens at different aging durations: (a) 112 h, (b) 225 h, (c) 337 h, and (d) 450 h.

Figure 7 shows the fracture images of the tensile specimens after different aging durations. When these specimens are compared among themselves, it is observed that the fractures occurred in the measurement region and displayed similar shapes. However, when evaluated together with the reference specimens (Figure 5), it can be seen that the aged specimens exhibited smoother fracture surfaces. This can be explained by the adverse effects of aging on the polymer structure, leading to embrittlement of the material.

**Figure 8:**
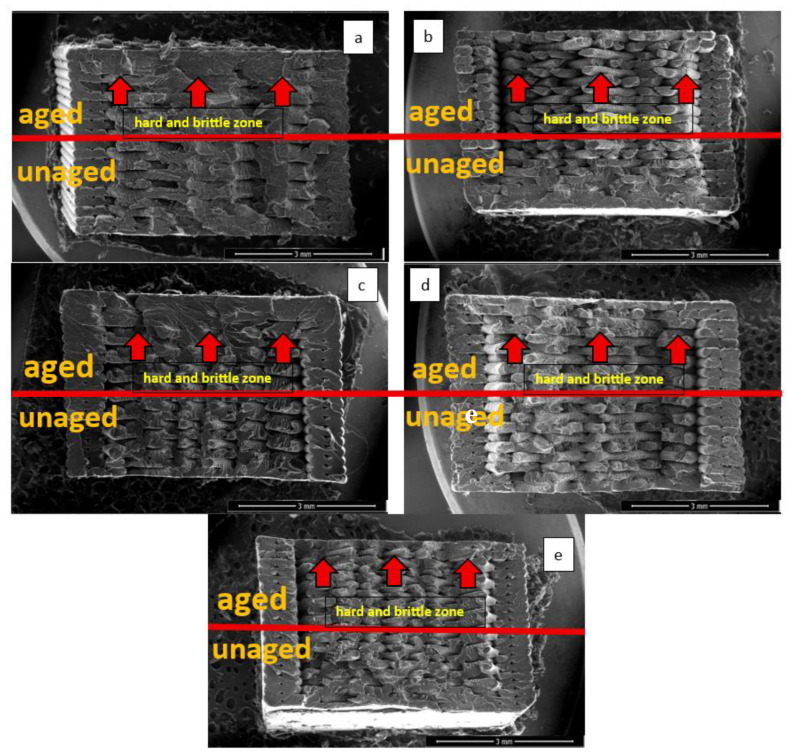
Fracture surface SEM images after aging: (**a**) red, (**b**) blue, (**c**) purple, (**d**) yellow, (**e**) green.

Figure 8 presents the scanning electron microscope (SEM) images of the fracture surfaces of the tensile test specimens after aging (a: green, b: red, c: purple, d: yellow, e: green). In the images, the upper part of each specimen represents the aged region, while the lower part corresponds to the unaged region. Examination of the SEM images revealed distinct differences between the surfaces exposed to xenon light and those that were not.

In all colors, the surfaces exposed to xenon light (particularly in the regions close to the outer surface) were observed to become embrittled and exhibit a more fragile structure. This can be attributed to changes in the chemical bond structures of the aged surfaces under the influence of radiation and temperature, resulting in a harder and more brittle morphology. In contrast, the surfaces not exposed to xenon light displayed ductile fracture behavior. In addition to radiation exposure, the repeated heating of the surfaces and their sudden cooling through spraying led to the formation of a hard and brittle structure extending from the outer surface toward the interior. In particular, as seen in Figure 8c, the purple specimens, due to the wavelength associated with their color, were more strongly affected and exhibited harder and more brittle behavior.

The observed increase in hardness, measured on the sample surface, is directly related to chain scission and cross-linking chemical processes, as well as the resulting changes in the polybutadiene (PB) phase. This local increase in hardness and stress hardening has been shown to cause brittleness and brittle behavior in the polymer. Therefore, it can be concluded that the increase in hardness serves as physical evidence of chain scission in the polybutadiene (PB) phase and surface degradation through cross-linking. The combination of these phenomena ultimately leads to the polymer becoming brittle [37,38,39,40].

### 3.3. Bending Results After Aging

**Figure 9:**
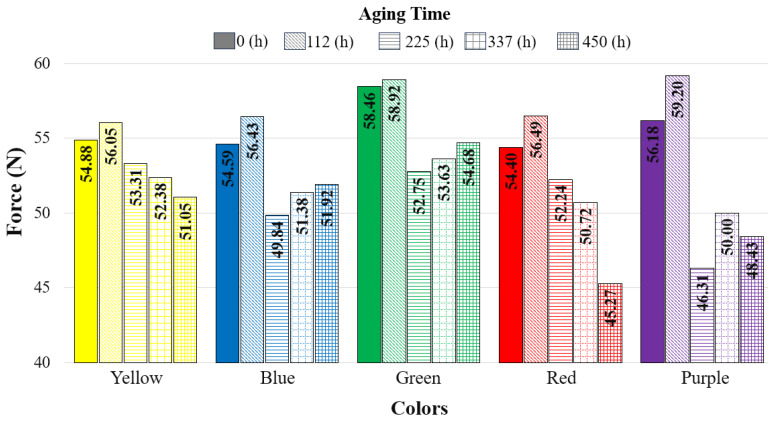
Comparison of bending forces with respect to aging duration.

Figure 9 presents the variation in bending forces with aging durations and filament colors, while Table 5 provides the corresponding force–displacement values. For all colors, the bending force increased after 112 h of aging. The bending force values for the purple, green, red, blue, and yellow specimens were determined as 59.20 N, 58.46 N, 56.49 N, 56.43 N, and 56.05 N, respectively, from highest to lowest. In the yellow and red specimens, the bending force decreased with longer aging durations (225, 337, and 450 h). In the blue and green specimens, a sharp decrease in bending force was observed after 112 h of aging, followed by an increase as aging duration progressed. For the purple specimens, the bending force sharply decreased at 225 h, increased again at 337 h, and then dropped once more.

The fracture surfaces of the bending specimens according to aging durations. It was observed that all aged test specimens bent at the loading point after the bending test but did not fracture completely. As the aging duration increased, the fracture appearance became more pronounced, and the cracks were observed to follow a straight-line path across the width of the specimens.

### 3.4. Hardness After Aging

**Figure 10:**
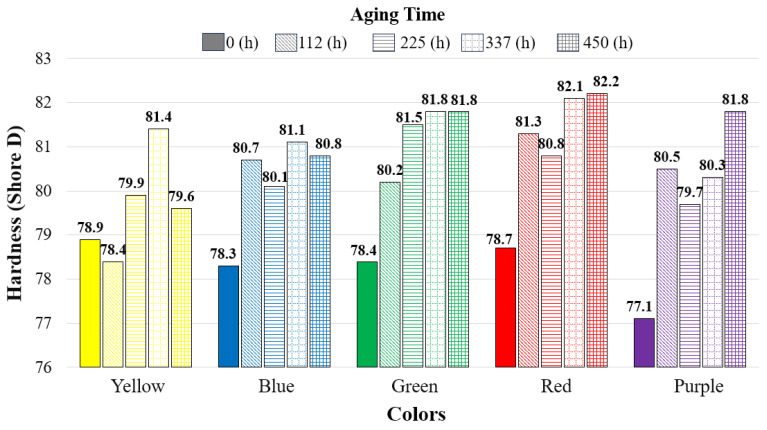
Comparison of hardness (Shore D) values with respect to aging duration.

Figure 10 presents the hardness values according to aging duration and color. In all specimens, hardness increased with aging duration compared to the reference specimens (except for the yellow specimens at 112 h of aging). The highest hardness increase was observed in the red specimens, where the hardness value increased from 78.7 Shore D (reference) to 82.2 Shore D after 450 h of aging. In addition, it was determined that the hardness of the green and red specimens increased consistently with aging duration.

### 3.5. Color and Gloss After Aging

In this study, the 3D-printed parts were first examined by visual inspection. Although the parts were produced from the same material, differences in surface gloss and matte appearance were observed. After aging, the total color change was found to increase with aging duration.

The greatest total color change values were measured in the specimens exposed to 450 h of aging. When all colors were evaluated together, the highest total color change was found in the purple specimens. When each color was assessed individually in comparison to its reference specimen, the largest color change was observed in the yellow specimens, while the smallest color change was observed in the green specimens. Table 6 presents the total color change of the specimens. These results also indicate an increase in the L* gloss value. An increase in the L* value suggests that the specimens began to fade and exhibit more gloss. This situation may be attributed not only to the degradation of ABS but also to the type of colorant additives used [22].

**Figure 11:**
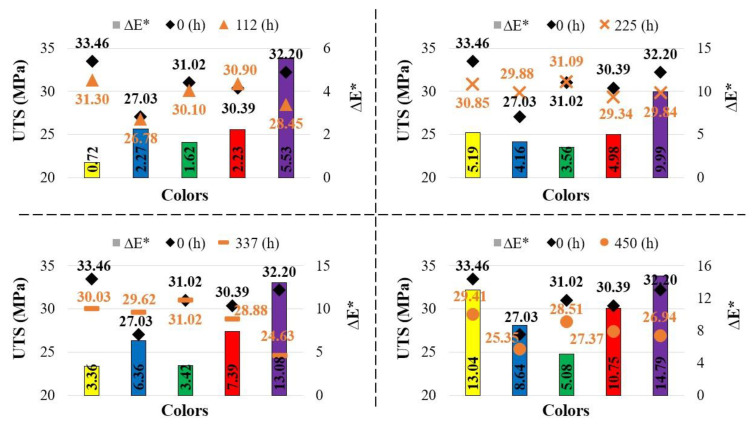
Relationship between total color change and UTS (ultimate tensile strength) (The values written on the columns indicate ${\Delta E}^{*}$).

Figure 11 shows the relationship between total color change and UTS (ultimate tensile strength). According to this graph, in the reference samples (0 h), the tensile strength, from highest to lowest, was found in the yellow (33.46 MPa), purple (32.20 MPa), green (30.10 MPa), red (30.39 MPa), and blue (27.03 MPa) samples.

The strength of the yellow and purple samples decreased depending on the aging duration. While the strength values measured after aging in the yellow samples were relatively close to each other, the decrease in the purple samples was much more pronounced. It was observed that the green samples reached higher strength than the blue samples and that their color changes were also less than those of the blue samples.

For the red samples, no significant difference in strength was observed at aging durations of 112 and 225 h, whereas a decrease in strength was noted at 337 and 450 h. In the green and blue samples, the effect of aging on strength became most evident at 450 h. Moreover, the strength increase–decrease trends of the blue and green samples depending on aging duration exhibited similar reactions.

**Figure 12:**
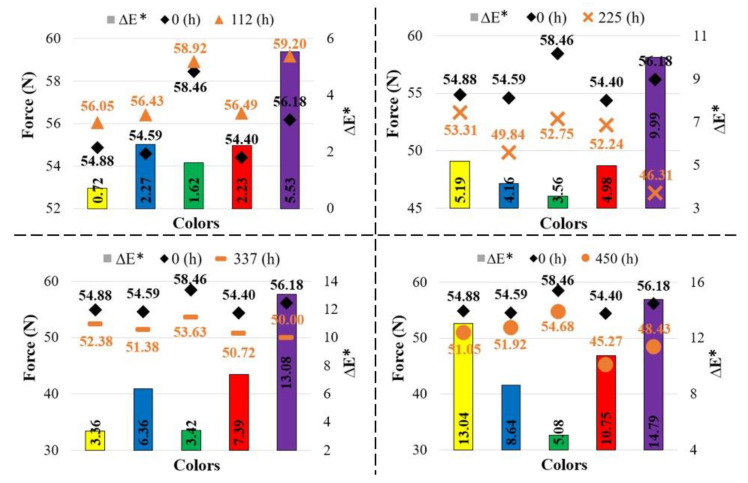
Relationship between color change and bending force (The values written on the columns indicate ${\Delta E}^{*}$).

Figure 12 presents the relationship between total color change and bending force. As can be seen from this graph, for the reference samples (0 h), the bending force, from highest to lowest, was measured in the green (58.46 MPa), purple (56.18 MPa), yellow (54.88 MPa), blue (54.59 MPa), and red (54.40 MPa) samples. When the reference materials were compared, the bending force of the samples increased at 112 h of aging; however, as the aging duration increased (225, 337, and 450 h), the bending force values of the samples decreased. The largest difference in bending force between the reference and aged samples was observed in the purple samples.

**Figure 13:**
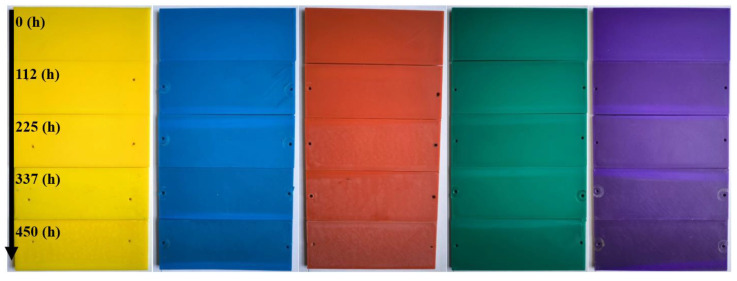
Surface appearances of the samples.

Figure 13 shows the surface appearances of the test samples before and after aging. It can be observed that there is a noticeable color change in the samples after aging. Since the gloss measurement values were below 10 GU, a measurement angle of 85° was adopted. These values are presented as the gloss of the samples. Based on the evaluation before and after aging, the purple samples were found to have the highest surface gloss.

The purple samples, which were determined to have the highest gloss, are also the parts most affected by the aging process. This result, consistent with the literature, indicates that the ABS material and pigment structure have begun to deteriorate [22].

Table 7 presents the gloss measurement values of the samples before and after aging. For the reference samples, gloss was found to range between 2.5 and 5.6 GU, whereas for the aged samples, it ranged between 2.5 and 7.7 GU. Gloss refers to the ability of a surface to reflect light from a specific angle, and the main factors affecting gloss are the surface roughness of the object and its molecular structure [41]. Pigments undergo changes under the influence of light. This change is related to the chemical structure, concentration, and physical state (particle size and structure) of the pigment. Therefore, the reflection properties of the surface vary depending on the different pigments present in the colored filaments [22]. In this study, it was observed that gloss values changed depending on the aging duration. Compared to the reference samples, the greatest changes in gloss were measured at aging durations of 337 and 450 h.

Fiorio et al. investigated the properties of ABS after aging by adding different masterbatches. The prepared samples were molded using plastic injection and then subjected to aging and subsequent recycling. Their experimental design included cyclic aging processes, and the mechanical and optical properties were measured after each operation. As a result, it was observed that the mechanical properties of the masterbatch-containing samples decreased after the first aging cycle. In subsequent aging cycles, the mechanical properties of the samples were reported to approach the values observed in the reference samples. Regarding the yellowing index of the parts, an increase was observed both after aging and following mechanical recycling. The authors attributed this change to polymer degradation [42].

In another study, it was found that as the aging duration of ABS plastics increased, both color change and absorbance increased. This was explained by the fact that aging-induced degradation primarily affects the surface of the material, with minimal chemical changes occurring within the material during aging. It was reported that as degradation progressed and absorbance increased, significant yellowing occurred. The observed degradation during aging was attributed to oxidation of the butadiene double bonds, formation of aldehydes or ketones, some chain scissions, and changes in the molecular weight of the ABS polymer [43]. These studies confirm that aging in polymers is accompanied by degradation and associated color changes.

Table 8 presents the surface roughness measurement results of the samples before and after aging. The surface roughness of the reference samples before aging ranged from 2.66 to 4.18 µm, whereas after aging, it was measured between 2.37 and 4.97 µm. The surface roughness of the purple samples increased markedly due to the effect of aging, while it decreased in the blue and yellow samples. In addition, the surface roughness of the green samples increased with aging, whereas in the red samples it tended to decrease.

In a study on the aging of ABS plates produced by injection molding [44], it was found that aging had a significant effect on the gloss and color of the parts. It was stated that the reduction in gloss as a result of artificial aging could be associated with a slight increase in the surface roughness of the samples. This study indicated that degradation might cause a change in the refractive index of the surface layers of the parts, which could in turn lead to a slight decrease in gloss level. In polymer parts produced by additive manufacturing, however, surface quality challenges differ considerably from those in conventional manufacturing. Poor surface quality and geometric deviations are often encountered. Nevertheless, there is no uniform standard for evaluating the roughness and dimensional accuracy of 3D-printed parts [45]. Sears found that the tensile strengths of polylactic acid (PLA) filaments of different colors (red, blue, yellow, and orange) varied. He also suggested that printing PLA filaments of different colors, even under the same printing settings, could affect print quality. One of the defining features of high-quality 3D printing is good surface quality. However, it is not always easy to identify the conditions affecting the surface quality of 3D-printed parts, since even parts produced with the same printing parameters and conditions may exhibit variations in surface roughness [46].

## 4. Conclusions

The variety and applications of plastic materials (e.g., daily use, marine, and automotive applications) continue to increase day by day. The growing use of plastics makes it necessary to investigate their behavior under different conditions. In the present study, ABS Plus materials in five different colors (yellow, green, blue, red, and purple) were produced using 3D printing. These specimens were subjected to aging under a xenon-arc lamp for 112, 225, 337, and 450 h.

In the reference specimens, the highest UTS was recorded in the yellow samples at 33.46 MPa, while the highest bending force was observed in the green samples at 58.46 N. This indicates that the additives used by manufacturers for coloring during filament production have a significant effect, and users should take this into consideration.

Among the aged specimens, the green color was the least affected up to 337 h. For the green specimens, the UTS values were determined as 31.02, 30.10, 31.09, and 31.02 MPa for the reference, 112, 225, and 337 h of aging, respectively. The wavelength of the green color lies between those of red and purple, which represent the longest and shortest wavelengths, respectively. The more stable change in the mechanical properties of the green specimens with respect to aging duration may be attributed to the more balanced distribution of heat during the aging process.

Each color of light has a different wavelength and exhibits distinct penetration and absorption properties [47]. The order of colors based on their ability to absorb solar heat, from highest to lowest, is black, green, red, purple, yellow, pink, blue, and white [48]. These studies show that color affects heating due to its wavelength. Another study indicates that infrared radiation is the primary wavelength responsible for generating heat, and therefore, the material’s absorption capacity significantly influences its heating [49]. Consequently, color can be considered an important factor in the aging of materials.

Examination of the bending test results revealed that, for all specimens, the values obtained after 112 h of aging were higher than those of the reference specimens. The green color exhibited more stable results in both tensile and bending tests. It was observed that the effects of aging duration on specimen properties varied depending on the specimen color.

Studies investigating the effects of aging demonstrated that color selection influences not only mechanical properties but also optical properties, indicating that both aspects should be taken into consideration when choosing filament color.

The content and amount of colorants added by filament manufacturers to commercial filaments are not precisely known. Therefore, it is difficult to determine the effects of these colorants on the final product and their relationship with the product’s performance under environmental conditions, such as UV exposure. It is expected that this issue will become clearer in the future as researchers developing filament materials include investigations of color and colorant structures in their studies.

## Figures and Tables

**Table 1:** ABS Plus mechanical properties [18].

Brand	eSUN
Filament	ABS Plus
Color	Yellow, Purple, Red, Green, Blue
Density (g/cm^3^)	1.06
Tensile Strength (MPa)	40
Elongation at Break (%)	30
Flexural Strength (MPa)	68
Izod Impact Strength (kJ/m^2^)	42

**Table 2:** UTS and elongation (%) values of the base materials.

Color	UTS (MPa)	Strain (%)
Yellow	33.46 ± 1.71	7.27 ± 0.23
Blue	27.03 ± 0.65	4.83 ± 0.82
Green	31.02 ± 0.96	5.83 ± 0.64
Red	30.39 ± 1.62	5.86 ± 1.01
Purple	32.20 ± 1.12	5.80 ± 0.71

**Table 3:** Force and displacement values of the base materials.

Color	Force (N)	Displacement (mm)
Yellow	54.88 ± 1.39	28.27 ± 1.42
Blue	54.59 ± 0.82	36.21 ± 2.13
Green	58.46 ± 0.49	28.55 ± 1.27
Red	54.40 ± 1.32	29.05 ± 1.59
Purple	56.18 ± 1.23	33.97 ± 2.03

**Table 4:** UTS and Strain of specimens at different aging times, with percentage change relative to 0 h reference.

Duration (h)	Color	Stress (MPa)	Strain (%)	Change Stress (%)	Change Strain (%)
112	Yellow	31.30 ± 1.82	6.02 ± 0.56	−6.46	−17.19
	Blue	27.78 ± 1.70	4.32 ± 0.19	2.77	−10.56
	Green	30.10 ± 1.26	4.62 ± 0.21	−2.97	−20.75
	Red	30.90 ± 0.96	5.15 ± 0.55	1.68	−12.12
	Purple	28.45 ± 1.43	3.86 ± 0.81	−11.65	−33.45
225	Yellow	30.85 ± 1.13	4.56 ± 0.54	−7.8	−37.28
	Blue	29.88 ± 1.96	4.28 ± 0.76	10.54	−11.39
	Green	31.09 ± 0.71	3.90 ± 0.46	0.23	−33.1
	Red	29.34 ± 1.91	4.51 ± 0.51	−3.46	−23.04
	Purple	29.84 ± 0.98	3.64 ± 0.29	−7.33	−37.24
337	Yellow	30.03 ± 1.74	3.26 ± 0.65	−10.25	−55.16
	Blue	29.62 ± 2.11	3.71 ± 0.53	9.58	−23.19
	Green	31.02 ± 1.81	3.80 ± 0.39	0.00	−34.82
	Red	28.88 ± 1.53	4.33 ± 0.26	−4.97	−26.11
	Purple	24.63 ± 1.26	3.14 ± 0.14	−23.51	−45.86
450	Yellow	29.41 ± 1.31	3.21 ± 0.31	−12.1	−55.85
	Blue	25.35 ± 1.91	3.43 ± 0.34	−6.22	−28.99
	Green	28.51 ± 1.17	3.01 ± 1.01	−8.09	−48.37
	Red	27.37 ± 0.92	3.47 ± 0.64	−9.94	−40.78
	Purple	26.94 ± 0.88	2.95 ± 0.48	−16.34	−49.14

**Table 5:** Force–displacement values at different aging durations.

Duration	112 (h)		225 (h)	
Color	Force (N)	Displacement (mm)	Force (N)	Displacement (mm)
Yellow	56.05 ± 1.28	16.52 ± 0.78	53.31 ± 1.33	15.58 ± 1.46
Blue	56.43 ± 1.96	28.26 ± 1.56	49.84 ± 1.02	16.07 ± 1.93
Green	58.92 ± 1.18	29.56 ± 2.13	52.75 ± 2.01	15.14 ± 0.66
Red	56.49 ± 0.92	24.22 ± 1.97	52.24 ± 1.73	13.58 ± 1.01
Purple	59.20 ± 1.53	28.17 ± 2.01	46.31 ± 1.89	13.11 ± 0.98
***Duration***	**337 (h)**		**450 (h)**	
***Color***	**Force (N)**	**Displacement (mm)**	**Force (N)**	**Displacement (mm)**
Yellow	52.38 ± 1.86	13.42 ± 0.98	51.05 ± 1.03	12.25 ± 1.02
Blue	51.38 ± 2.01	15.33 ± 1.21	51.92 ± 1.98	14.78 ± 0.73
Green	53.53 ± 0.86	15.08 ± 1.13	54.68 ± 1.66	15.91 ± 1.55
Red	50.72 ± 1.02	13.37 ± 0.76	45.27 ± 0.78	11.97 ± 0.45
Purple	50.00 ± 1.53	13.83 ± 0.85	48.43 ± 1.03	10.72 ± 0.63

**Table 6:** Total color change of the specimens.

Color		Reference	Aging Duration (h)			
			112	225	337	450
Yellow	L*	81.52	82.20	81.80	82.05	81.66
	a*	1.98	2.14	3.53	−1.30	3.53
	b*	75.91	75.78	70.96	76.37	62.97
${\Delta E}^{*}$			0.72	5.19	3.36	13.04
Purple	L*	35.01	34.07	36.21	36.98	37.01
	a*	23.81	20.53	17.65	15.97	14.82
	b*	−32.19	−27.84	−24.43	−21.91	−20.62
${\Delta E}^{*}$			5.53	9.99	13.08	14.79
Red	L*	47.30	47.22	48.17	48.43	50.11
	a*	42.93	41.40	39.35	37.67	34.99
	b*	24.12	22.49	20.77	19.05	17.44
${\Delta E}^{*}$			2.23	4.98	7.39	10.75
Green	L*	51.84	50.56	49.43	50.17	48.69
	a*	−38.72	−38.25	−36.25	−36.25	−34.86
	b*	5.84	6.72	6.71	7.51	6.84
${\Delta E}^{*}$			1.62	3.56	3.42	5.08
Blue	L*	43.54	42.73	43.18	42.01	41.89
	a*	−13.81	−14.21	−15.60	−15.35	−15.26
	b*	−27.18	−25.10	−23.45	−21.21	−18.83
${\Delta E}^{*}$			2.27	4.16	6.36	8.64

**Table 7:** Gloss values of the specimens.

Color	Geometries (Angles)	Reference	Aging Duration (h)			
			112	225	337	450
Yellow	20°	0.6	0.6	0.6	0.7	0.7
	60°	3.7	3	3	4.6	3.7
	85°	2.5	2.6	3	3.5	3.7
Purple	20°	0.0	0.0	0.0	0.1	0.1
	60°	3.0	3.4	3.8	4.3	4.9
	85°	5.6	6.7	7.0	7.7	7.9
Red	20°	0	0	0	0	0
	60°	2.2	2	1.9	2.1	2
	85°	3.1	3	3.1	3.2	3.1
Green	20°	0	0	0.1	0.1	0.1
	60°	2.3	2.7	3.6	3.2	4.5
	85°	4.7	6.3	4.6	6.4	5.7
Blue	20°	0	0	0	0	0.1
	60°	2.9	3.5	2.9	3.4	3.9
	85°	5.4	2.7	5.3	6.8	6.6

**Table 8:** Arithmetic mean surface roughness (Ra) values (µm) of the samples.

Color	Surface Roughness (µm)				
	Reference	112 h	225 h	337 h	450 h
Yellow	4.2	2.9	4.2	4.0	3.5
Purple	3.7	4.5	3.9	4.6	4.5
Red	4.8	3.6	4.9	4.1	3.4
Green	2.7	3.6	4.6	2.4	3.9
Blue	4.3	3.5	2.4	3.6	3.0

## Data Availability

The original contributions presented in this study are included in the article. Further inquiries can be directed to the corresponding author.
